# A single electrical pulse within the protective zone of each cardiac cycle prevented reperfusion‐induced ventricular tachycardia in conscious mice

**DOI:** 10.14814/phy2.13578

**Published:** 2018-01-22

**Authors:** Heidi L. Lujan, Joshua P. Rivers, Stephen E. DiCarlo

**Affiliations:** ^1^ Department of Physiology College of Osteopathic Medicine Michigan State University East Lansing Michigan; ^2^ Wayne State University School of Medicine Detroit Michigan

**Keywords:** Arrhythmia, cardioprotection, electrocardiogram, murine model

## Abstract

Early pioneering investigators discovered, in anesthetized dogs, a protective period within the cardiac cycle. The protective period was a time within the cardiac cycle when a precisely timed stimulus prevented the initiation of ventricular fibrillation caused by an earlier stimulus. Thus, in addition to the susceptible period of repolarization discussed by Wiggers and Wegria (Am. J. Physiol. 131:296, 1940; Am. J. Physiol. 128:500, 1940), there is also a nearby protective period. This report describes a protective period within the cardiac cycle of conscious mice when a precisely timed stimulus prevented the initiation of ventricular tachycardia caused by an earlier stimulus. In addition, we tested the hypothesis that this precisely timed pulse within the protective period prevents reperfusion‐induced ventricular tachyarrhythmias in conscious mice. Mice (*n *=* *6) were prepared to record arterial blood pressure and the electrocardiogram. In addition, a vascular occluder was placed around the left main coronary artery, and stimulating electrodes were secured onto the left ventricle. A single precisely timed electrical pulse (5 msec pulse width and 2.5 V) to the left ventricle arriving 13.9 ± 1.1 msec after the R‐wave, caused ventricular tachycardia occurring 24.9 ± 0.9 msec after the R‐wave. Importantly, a second precisely timed electrical pulse arriving 18.8 ± 0.5 msec after the first stimulus blocked the induction of ventricular tachycardia caused by the earlier stimulus. On an alternate day, the susceptibility to sustained ventricular tachycardia produced by 3.5 min of occlusion and reperfusion of the coronary artery was determined in conscious mice by use of the vascular occluder. Reperfusion resulted in ventricular tachycardia in all six mice. A precisely timed pulse within the protective period prevented ventricular tachycardia in all mice.

## Introduction

The use of electrical currents to restore homeostasis is an emerging field. As examples, cardiac pacemakers and defibrillators restore cardiac rhythms, deep‐brain stimulation improves motor control for individuals living with Parkinson's disease, sacral nerve stimulation improves bladder control in individuals living with spinal cord injury, and vagal nerve stimulation is useful for individuals living with heart failure, epilepsy, and rheumatoid arthritis.

Electrical impulses have also been used to prevent cardiac rhythm disorders (Durrer et al. 1967; Hunt et al. 1968; Barold et al. 1969; Wellens et al. 1972). Specifically, investigators have described a period within the cardiac cycle during which a precisely timed stimulus can block the induction of ventricular fibrillation caused by a previous stimulus (Tamargo et al. 1975; Verrier et al. 1978). This precisely timed stimulus within the protective zone of the cardiac cycle has been theorized to cause its anti‐arrhythmic effects by blocking local reentrant activity caused by the previous stimulus (Euler and Moore 1980).

These early pioneering studies were conducted in anesthetized dogs (Verrier and Lown 1982), and ventricular fibrillation was induced by nonphysiological procedures. Accordingly, it is unknown if a protective zone exists in conscious murine models or if the protective zone can prevent ventricular tachyarrhythmias induced by a clinically relevant stimulus. Thus, this study was designed to determine whether a precisely timed single pulse within the protective zone of the cardiac cycle can block the induction of ventricular tachyarrhythmias induced by an earlier stimulus in conscious mice (i.e., does a protective zone exist in a conscious murine model?) In addition, we tested the hypothesis that a precisely timed single pulse within the protective zone of each cardiac cycle blocks reperfusion‐induced ventricular tachyarrhythmias induced by 3.5 min of occlusion and reperfusion of the left main coronary artery.

## Materials and Methods

### Experimental subject

Experimental and surgical approaches were studied and approved by the Institutional Animal Care and Use Committee and followed the American Physiological Society's Guiding Principles in the Care and Use of Vertebrate Animals in Research and Training. Studies examining the protective period and the susceptibility to reperfusion‐induced ventricular tachyarrhythmias were performed in six adult male C57BL/6J mice (15 weeks of age), a strain frequently utilized in transgenic studies (Berul et al. 1996).

### Surgical Procedures

#### Instrumentation

Mice were anesthetized with sodium pentobarbital (90 mg/kg, i.p.) and prepared for aseptic surgery. Additional pentobarbital (10–20 mg/kg, i.p.) was administered if the mice responded to tail pinch.

**Figure 1:**
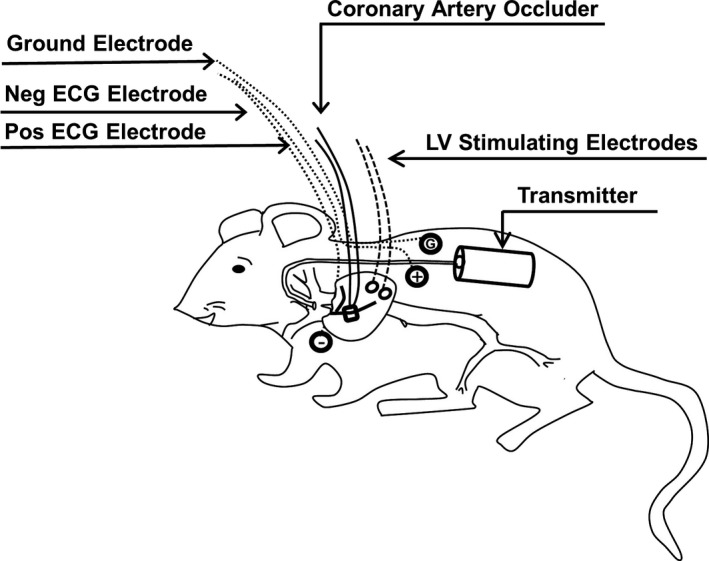
Presents a schematic representation of a mouse chronically instrumented with a PA‐C10 transmitter, left ventricular‐stimulating electrodes, coronary artery occluder, positive (pos) and negative (neg) electrocardiogram electrodes, and a ground (G) electrode.

A left thoracotomy through the second intercostal space exposed the heart, and Teflon‐coated stainless steel wires were secured onto the surface of the left ventricle as previously described in mice (Lujan and DiCarlo 2014a; Lujan et al. 2016). The stimulating wires were passed subcutaneously and exited at the dorsal aspect of the neck. Next, a coronary artery occluder was passed around the left main coronary artery as recently described in mice (Lujan et al. 2012; Lujan and DiCarlo 2013, 2014b, 2017). The two ends of the vascular occluder were passed through guide tubing and exited at the dorsal aspect of the neck (Lujan et al. 2012; Lujan and DiCarlo 2013, 2014b, 2017). Subsequently, to record arterial blood pressure, a catheter from a telemetry device (Data Sciences International, PA‐C10) was placed into the left carotid artery and advanced to the aortic arch (Kurtz et al. 2014; Lujan and DiCarlo 2017). The transmitter body was positioned subcutaneously on the left side. Finally, ECG electrodes were positioned subcutaneously in a modified lead II configuration, passed subcutaneously, and exited at the dorsal aspect of the neck as previously described in mice (Lujan and DiCarlo 2013; Lujan and DiCarlo 2013; Lujan and DiCarlo 2017; Lujan and DiCarlo 2014a,b; Lujan et al. 2012; Lujan et al. 2016). A schematic presenting the surgical preparation is shown in Figure 1. Mice received preoperative analgesics bupivacaine (1 mg/kg, sq) at all incision sites and carprofen (5 mg/kg, sq). Buprenorphine (0.1 mg/kg, sq) and carprofen were given for 2 days during postoperative care. To avoid acute postoperative infections, cefazolin (10 mg/kg, sq) was administered preoperatively and postoperatively for 2 days. Ten days were allowed for recovery when the mice were familiarized to the laboratory and investigators.

### Determination of the Vulnerable Period

Mice were studied in the conscious and unrestrained condition in their standard home cages during the light cycle for all experiments. The temperature within the cage was maintained at the thermoneutral zone for mice of approximately 29–31°C (Swoap et al. 2004). Mice adapted to the experimental environment for approximately 2 h to ensure steady hemodynamic conditions.

Following the adaptation period, steady‐state heart rate, arterial blood pressure, and ECG parameters were recorded over 10–15 sec. Next, the vulnerable period within the cardiac cycle that caused ventricular tachycardia was determined. Specifically, the ECG was monitored and directed to a window discriminator. The window discriminator was instrumented with a switch that permitted the R‐wave to activate a Grass SD9 stimulator, sending one precisely timed pulse through the ventricular stimulating electrodes. The delay on the stimulator permitted the R‐wave stimulus interval to be adjusted. The vulnerable period represented the R‐wave stimulus interval causing ventricular tachycardia.

To determine the protective period, the stimulus from the SD9 stimulator that caused ventricular tachycardia was also sent to activate a second SD9 stimulator. The delay on the second stimulator was adjusted to find the period within the cardiac cycle that blocked the first stimulus from causing ventricular tachycardia. Specifically, the protective period was the time within the cardiac cycle during which a precisely timed stimulus prevented the induction of ventricular tachycardia caused by the earlier stimulus.

### Susceptibility to reperfusion‐induced sustained ventricular tachycardia

The order of the next two protocols, control and intervention, was randomized. For the control experiments, the coronary artery was temporarily occluded for 3.5 min in conscious mice as previously described (Lujan and DiCarlo 2014b). Standard changes in the ECG (peaked T‐wave and ST‐segment elevation) and a fall in arterial blood pressure occurred within seconds of coronary artery occlusion (Lujan et al. 2012; Lujan and DiCarlo 2014b, 2017). Upon release, all animals sustained ventricular tachycardia. Normal sinus rhythm appeared by gently compressing the thorax or in some cases required person to mouse ventilation via a customized tube over the nose and mouth. Without intervention, the sustained ventricular tachycardia progresses to ventricular fibrillation (VF).

On a separate day, for the intervention protocol, the ischemia–reperfusion protocol was conducted with a precisely timed single electrical pulse within the protective zone of each cardiac cycle. Specifically, the ECG was monitored and directed to a window discriminator. The window discriminator was instrumented with a switch that permitted the R‐wave to activate a Grass SD9 stimulator, sending one precisely timed pulse through the ventricular‐stimulating electrodes. The delay on the stimulator permitted the R‐wave stimulus interval to be adjusted so that the electrical impulse arrived within the protective zone of the cardiac cycle. Specifically, the R‐wave stimulus interval was set so that the stimulus fell within the protective period of the cardiac cycle.

### Data analysis

A sampling frequency of 4 kHz was used for all physiological recordings, and data were expressed as means ± SE. A one‐way analysis of variance with repeated measures was used to compare the arterial pressure and heart rate responses before stimulation in the vulnerable period (prestimulation), during the stimulation within the vulnerable period causing ventricular tachycardia (ventricular tachycardia), and following the cessation of ventricular tachycardia (recovery). A Holm–Sidak post hoc analysis was used to isolate group differences. A Student's paired *t*‐test was used to compare arterial pressure and heart rate before stimulation in the protective period (prestimulation) and following stimulation in the protective period (recovery). An alpha level of 0.05 was used to determine statistical significance.

Finally, box and whisker plots of the vulnerable and protective zones, arterial pressure and heart rate before the stimulus (which caused ventricular tachycardia) and during and after ventricular tachycardia as well as before the stimulus (which did not cause tachycardia), and following the stimulus were generated to display the variation in the data. The central box represents the values from the first and third quartiles (25–75 percentile). The vertical lines (whiskers) denote minimum and maximum values. The bold center line in each box plot represents the median value.

## Results

### Determination of the vulnerable zone within the cardiac cycle

**Figure 2:**
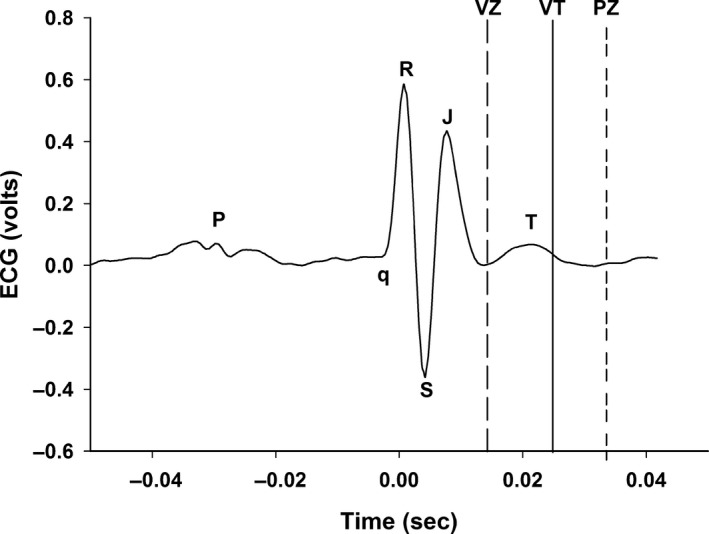
Presents an original recording of one electrocardiogram complex. Note that in the mouse the ST segment is not isoelectric and has a characteristic J‐wave that represents early repolarization (Boukens et al. 2013). The vulnerable zone was a period within the cardiac cycle during which a precisely timed single electrical impulse caused ventricular tachycardia. The protective zone was a period within the cardiac cycle during which a precisely timed stimulus blocked the induction of ventricular tachycardia caused by an earlier stimulus within the vulnerable zone.

**Figure 3:**
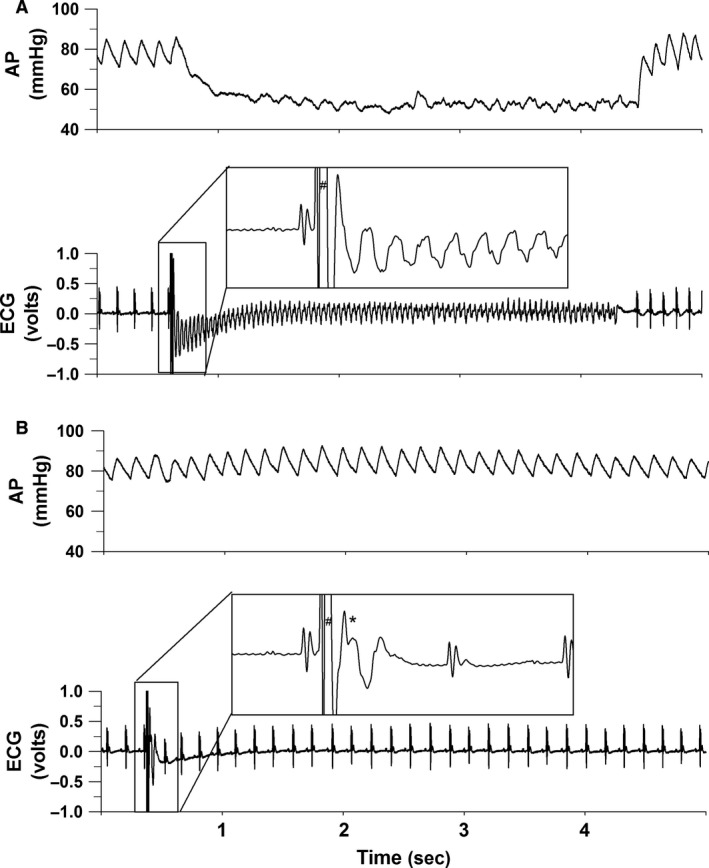
Panel (A) presents arterial blood pressure and the electrocardiogram in a mouse where a precisely timed single electrical pulse (#, *inset*) was within the vulnerable zone of the cardiac cycle and caused ventricular tachycardia. Ventricular tachycardia was identified on the electrocardiogram as rapid, wide QRS complexes with concomitant fall in arterial blood pressure. In Panel B, a second precisely timed electrical pulse (*, *inset*) was within the protective zone of the cardiac cycle and blocked the induction of ventricular tachycardia caused by the earlier stimulus.

The vulnerable zone (VZ, Fig. 2) was a period within the cardiac cycle during which a precisely timed single electrical pulse provoked ventricular tachycardia (Fig. 3, Panel A). Ventricular tachycardia was defined as the absence of P‐wave, wide QRS complex, a 300 ± 32 bpm increase in heart rate with a 17 ± 6 mmHg fall in arterial pressure. Normal sinus rhythm appeared spontaneously. That is, a precisely timed single electrical pulse (5 msec pulse width and 2.5 V) to the left ventricle arriving 13.9 ± 1.1 msec after the R‐wave caused ventricular tachycardia (VT) occurring 24.9 ± 0.9 msec after the R‐wave (Figs. 2 and 3, Panel A). The vulnerable zone was found in all six mice.

**Figure 4:**
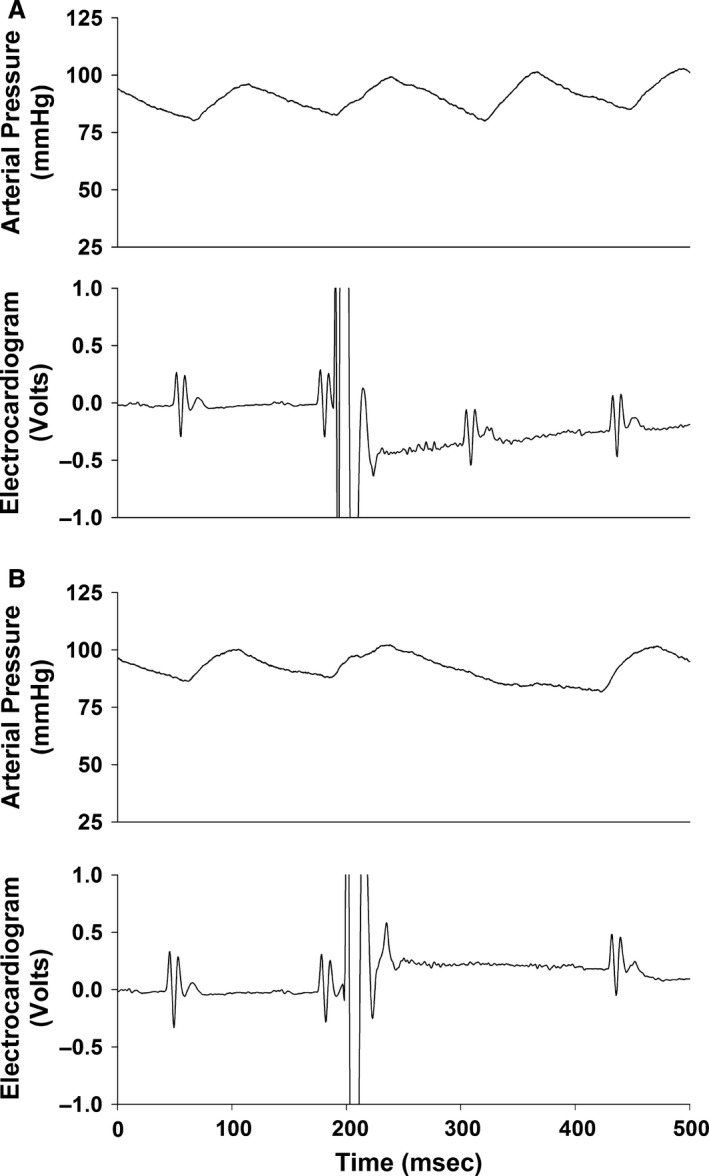
Presents 0.5 sec analog recordings of arterial pressure and the electrocardiogram with a precisely timed stimulus arriving early within the refractory period (11.75 msec after the R‐wave; Panel A) and outside the refractory period (19.75 msec after the R‐wave; Panel B). A pulse arriving within the refractory period failed to evoke a cardiac response (Panel A). A pulse arriving outside the refractory period depolarized the heart and elicited a compensatory pause but failed to cause ventricular tachycardia (Panel B). The Y scale on the electrocardiogram figures was expanded to display the details of the electrocardiogram and resulted in the stimulus shown off scale.

The timing of the pulse within the vulnerable zone to cause ventricular tachycardia was critical. For example, a pulse arriving early within the refractory period failed to evoke a cardiac response (Fig. 4 panel A). Furthermore, a pulse arriving outside the refractory period depolarized the heart, elicited ventricular depolarization, and a compensatory pause but failed to cause ventricular tachycardia (Fig. 4 panel B).

**Figure 5:**
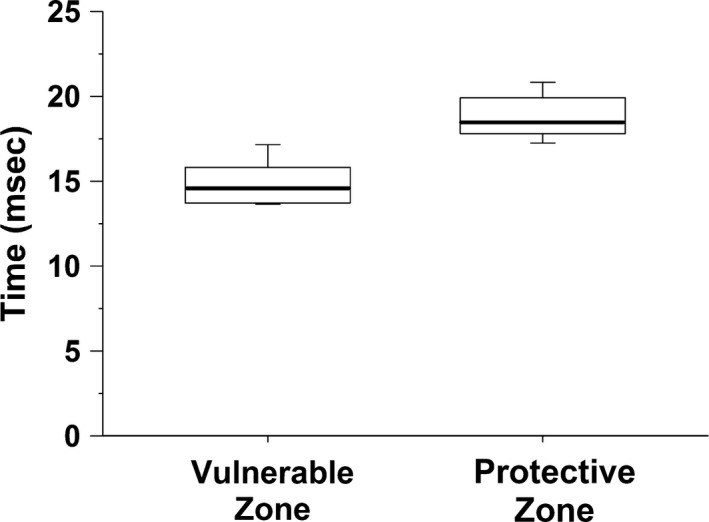
Box plot of the vulnerable and protective zone. The vulnerable zone is a period within the cardiac cycle during which a precisely timed single electrical impulse caused ventricular tachycardia. The protective zone is a period within the cardiac cycle during which a precisely timed stimulus blocked the induction of ventricular tachycardia caused by an earlier stimulus within the vulnerable zone. The central box represents the values from the first and third quartiles (25–75 percentile). The vertical lines (whiskers) denote minimum and maximum values. The bold center line in each box plot represents the median value.

A protective zone (PZ, Fig. 2) was a period within the cardiac cycle during which a precisely timed stimulus prevented the induction of ventricular tachycardia (VT) caused by an earlier stimulus within the vulnerable zone. The protective zone within the cardiac cycle that prevented the stimulus within the vulnerable period from inducing ventricular tachycardia averaged 18.8 ± 0.5 msec after the first stimulus (Fig. 2 and 3, Panel B). The protective zone was found in all six mice. The data distribution of the vulnerable and protective zones is presented as box and whisker plots (Fig. 5).

### Hemodynamics during determination of the vulnerable period and protective zone

**Figure 6:**
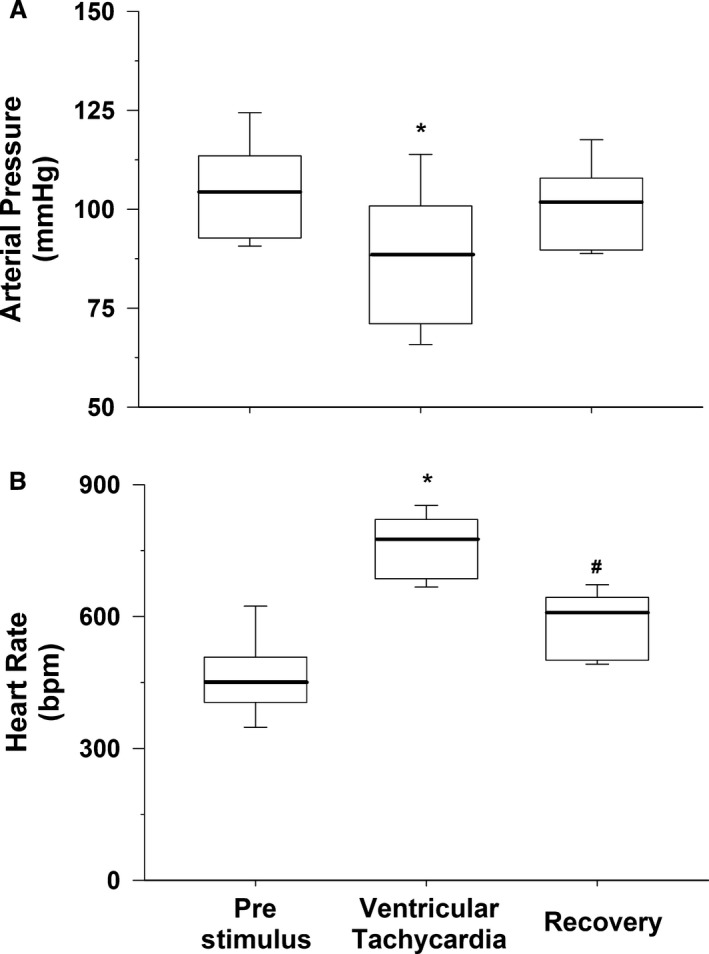
Box plots of arterial pressure (Panel A) and heart rate (Panel B) before the stimulus, after the stimulus which induced ventricular tachycardia (Ventricular Tachycardia), and following ventricular tachycardia (Recovery). The central box represents the values from the first and third quartiles (25–75 percentile). The vertical lines (whiskers) denote minimum and maximum values. The bold center line in each box plot represents the median value. *P < 0.05, Prestimulus versus Ventricular Tachycardia; #*P* < 0.05, Prestimulus versus Recovery

The electrical pulse within the vulnerable zone significantly increased heart rate from 462 ± 37 to 762 ± 30 bpm. The ventricular tachycardia was associated with a significantly reduced arterial pressure from 105 ± 5 to 88 ± 7 mmHg. The data distribution of arterial pressure and heart before the stimulus (Prestimulus), during the stimulus within the vulnerable period causing ventricular tachycardia, and following the cessation of ventricular tachycardia (recovery) is presented as box and whisker plots (Fig. 6).

**Figure 7:**
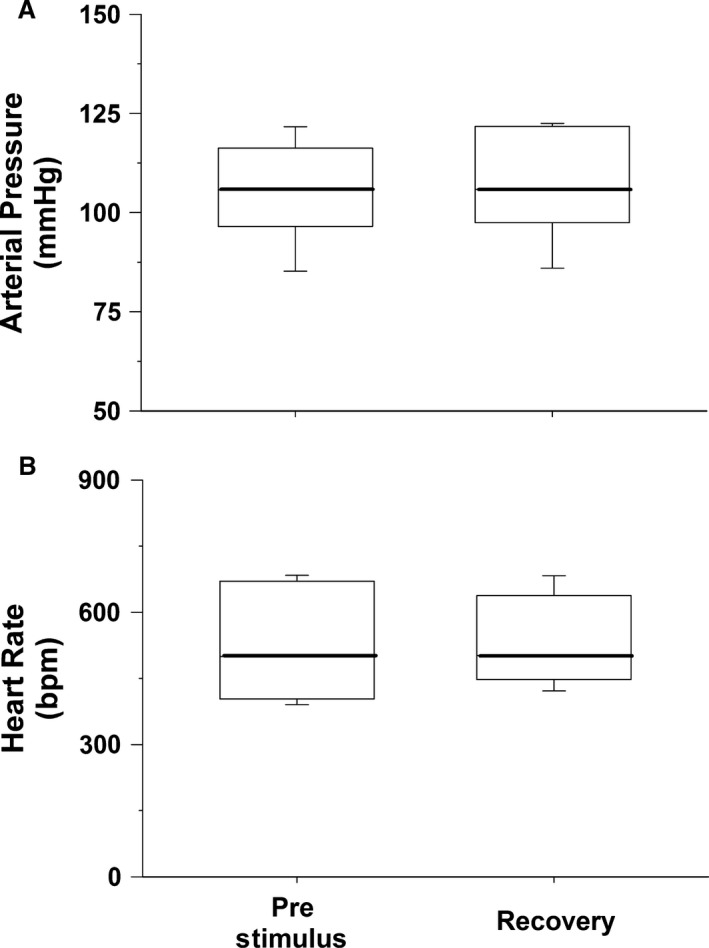
Box plots of arterial pressure (Panel A) and heart rate (Panel B) before (Prestimulus) and after (Recovery) the stimulus which did not cause ventricular tachycardia. The central box represents the values from the first and third quartiles (25–75 percentile). The vertical lines (whiskers) denote minimum and maximum values. The bold center line in each box plot represents the median value.

The precisely timed stimulus within the protective zone did not significantly change arterial pressure (105 ± 5 vs. 107 ± 5 mmHg) or heart rate (529 ± 60 vs. 534 ± 46 bpm). The intensity and duration of each electrical impulse within the protective zone that prevented ventricular tachycardia were 0.5 V and 5 msec duration, respectively. The data distribution of arterial pressure and heart rate before the stimulus (Prestimulus) and after the stimulus in the protective zone (recovery) is presented as box and whisker plots (Fig. 7).

**Figure 8:**
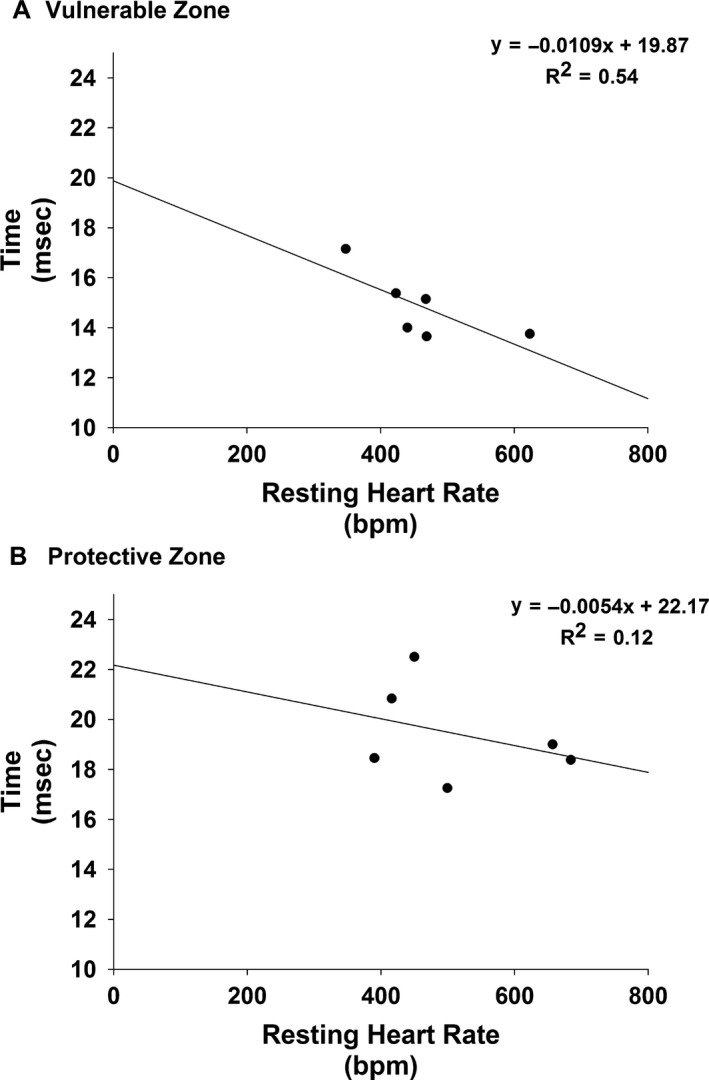
Presents the relationship between the vulnerable zone and heart rate (Panel A) and the protective zone and heart rate (Panel B) during the two protocols. Linear regression analysis revealed that heart rate was significantly correlated with the vulnerable zone; however, the relation was not as strong during determination of the protective zone.

Figure 8 presents the relationship between the vulnerable zone and heart rate (Panel A) and protective zone and heart rate (Panel) during the two protocols.

### Stimulation within the protective zone of the cardiac cycle prevented reperfusion‐induced ventricular tachycardia

**Figure 9:**
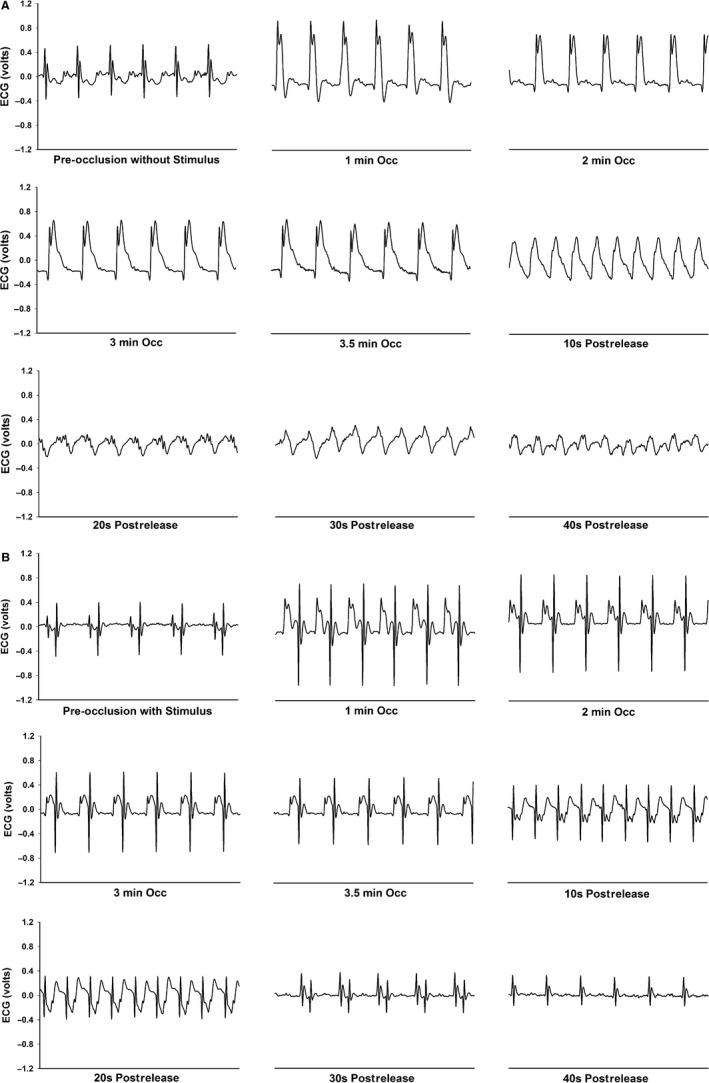
Presents original recordings of the ECG during the ischemia‐reperfusion protocol without (Panel A) and with a precisely timed single electrical pulse within the protective zone of each cardiac cycle (Panel B). Each panel presents 0.5 sec of the ECG before occlusion (without stimulus; Panel A and with stimulus; Panel B), at 1, 2, 3, and 3.5 min of occlusion, and at 10, 20, 30, and 40 sec after occlusion (Postrelease). Acute coronary artery occlusion induced rapid changes in the ECG (peaked T‐wave and ST‐segment elevation, (Panel A). Upon reperfusion, all animals experienced ventricular tachycardia (Panel A). On an alternate day (Panel B), coronary artery occlusion was repeated with a precisely timed single electrical pulse within the protective zone of each cardiac cycle. The precisely timed single electrical pulse within the protective zone blocked reperfusion‐induced ventricular tachycardia in every animal (Panel B). ECG, electrocardiogram.

The susceptibility to sustained ventricular tachycardia produced by 3.5 min of occlusion and reperfusion of the coronary artery was determined in conscious mice. Reperfusion culminated in ventricular tachycardia in all six mice (Fig. 9, Panel A). A precisely timed electrical impulse within the protective zone of each cardiac cycle prevented ventricular tachycardia in all six mice (Fig. 9, Panel B). The intensity and duration of each electrical impulse within the protective zone that prevented ventricular tachycardia were 0.5 V and 0.5 msec duration, respectively.

## Discussion

Cohnheim and Schulthess‐Rechberg (1881) originally observed ventricular fibrillation following reperfusion of a coronary artery. Subsequently, Tennant and Wiggers (1935) confirmed these observations. Later, it became clear that most individuals effectively resuscitated from sudden ventricular fibrillation related to coronary artery disease do not develop a myocardial infarction (Cobb et al. 1980; Goldstein et al. 1981; Wit and Janse 2001). This realization suggested that reperfusion may have occurred in some cases and provoked the ventricular fibrillation. Although ischemia is a more common trigger of sudden death than is reperfusion, reperfusion‐induced lethal ventricular arrhythmias are also associated with unstable angina, exercise‐induced ischemia, coronary artery vasospasm, and silent ischemia (Previtali et al. 1983; Myerburg et al. 1992; Lie 1993). Thus, despite the fact that little or no tissue damage happens during the brief periods of ischemia, reperfusion can cause lethal ventricular arrhythmias (Leary 1934; Prchkov et al. 1974; Maseri et al. 1978, 1982; Kerin et al. 1979; Myerburg et al. 1992; Sanna et al. 2009) that can lead to sudden cardiac death (Manning and Hearse 1984; Van Wagoner and Bond 2001). As sudden cardiac death due to coronary artery occlusion is a principle cause of death worldwide (Myerburg and Castellanos 1997), this is a significant problem for which there are few preventive measures.

In this study, for the first time, we documented a vulnerable zone and a protective zone (Wiggers and Wegria, 1940a; Wiggers and Wegria, 1940b) within the cardiac cycle of complex conscious mice. In addition, we documented that a precisely timed single electrical pulse within the protective zone of each cardiac cycle blocks ventricular tachycardia induced by myocardial ischemia and reperfusion in complex conscious mice. The possibility that stimulation within the protective zone is a safe and effective preventive measure for cardiac rhythm disorders merits further investigation.

Specifically, reperfusion‐induced ventricular fibrillation is notoriously drug‐resistant, most likely due to it being initiated by multifocal automaticity within the reperfused zone that is maintained by complex reentry in a changing landscape of electrophysiological substrates. In contrast, ischemia‐induced ventricular fibrillation is triggered by the flow of the injury current and/or reentry and maintained by reentry. An intervention that ameliorates the complex mechanisms of reperfusion‐induced ventricular fibrillation may also protect against the less complex ischemia‐induced ventricular fibrillation. Thus, this study supports the concept that a precisely timed single electrical pulse within the protective zone of each cardiac cycle has practical protective potential for ischemia‐induced ventricular fibrillation. However, it is important to note that it may be easier to terminate ventricular fibrillation in a rat or mouse heart than in a larger human heart where there are more reentry circuits in ventricular fibrillation.

The vulnerable zone likely represents a time of repolarization inhomogeneity when some cells are absolutely refractory, other cells are relatively refractory, and some cells have completely recovered excitability (Rossi et al. 2014). Thus, in this inhomogeneous environment, the refractory cells create a zone of unidirectional conduction block and slowly conduction zones that cause the electrical pulse to initiate reentrant excitation (Rossi et al. 2014). Conditions that increase inhomogeneity of refractoriness or repolarization are linked to a greater probability for reentrant excitation. Importantly, mice may be particularly susceptible because the QRS complex is composed of ventricular depolarization as well as early repolarization and the ST segment is not isoelectric, having a characteristic J‐wave that represents early repolarization (Boukens et al. 2013) and perhaps an increased inhomogeneity of refractoriness.

Programed electrical stimulation across the vulnerable zone of the cardiac cycle is widely used for assessing vulnerability to ventricular fibrillation (Han 1969; Moore and Spear 1975; Zipes 1975). However, the closely located protective zone may significantly affect results by providing a degree of protection, and thus, variability (Lown and Verrier 1976). However, inducing ventricular tachycardia arrhythmias with a precisely timed single pulse within the vulnerable zone (Wiggers and Wegria, 1940a; Wiggers and Wegria, 1940b) may provide a method to overcome this variability and be of importance for advancing the concepts and methods that drive anti‐arrhythmic therapies.

A precisely timed single pulse within the protective zone of the cardiac cycle has been theorized to exert its anti‐arrhythmic effects by blocking local reentrant activity caused by the previous stimulus (Euler and Moore 1980). Reentry is a major mechanism of cardiac arrhythmias. Reentry is a self‐sustained propagating activation front within an excitable tissue (Wit and Cranefield 1978). Programed stimulation, where one or more stimuli are applied within the reentry circuit, is used to block ventricular and atrial tachyarrhythmias (Gardner et al. 1982; Rosenthal et al. 1986). The mechanism mediating the anti‐arrhythmic effect is believed to be a unidirectional block. Specifically, the single pulse arrives during the critical period of the propagating waveform and produces a backward front that collides with the reentrant activity (Wiener and Rosenblueth 1946; Glass and Josephson 1995).

This is an important consideration because reperfusion‐induced arrhythmias are also likely mediated by an increased inhomogeneity in repolarization in and around the previous ischemic zone thus enhancing the likelihood for reentry (Janse 1982). The source of the original ectopic beat that causes the arrhythmia may be close to the border but are not triggered by reentry (Janse 1982). Accordingly, the precisely timed stimulus within the protective zone of the cardiac cycle likely exerts its anti‐arrhythmic effects by blocking local reentrant activity induced by the reperfusion.

Programed electrical stimulation protocols are used clinically and experimentally to obtain data about the cardiac conduction system and guide in the treatment of heart rhythm disorders. Specifically, programed electrical stimulation is used to understand mechanisms of ventricular tachycardia as well as the effect of pharmacological agents on arrhythmia mechanisms. The procedures used in this study conducted on conscious C57BL/6J mice can be utilized to enhance our understanding of mechanisms and treatments for reperfusion‐induced lethal ventricular arrhythmias in intact, conscious, and complex animals.

## Conflict of Interest

The author(s) declare(s) that there is no conflict of interest.
